# Passive Smoking Is Associated with Poor Asthma Control during Pregnancy: A Prospective Study of 500 Pregnancies

**DOI:** 10.1371/journal.pone.0112435

**Published:** 2014-11-19

**Authors:** Pernille A. Grarup, Julie H. Janner, Charlotte Suppli Ulrik

**Affiliations:** 1 Department of Pulmonary Medicine, Hvidovre Hospital, Hvidovre, Denmark; 2 University of Copenhagen, Copenhagen, Denmark; University of Athens Medical School, Greece

## Abstract

**Background and Aim:**

Asthma and tobacco exposure is common among pregnant women. We investigated the effect of passive and active smoking on asthma control during pregnancy.

**Methods:**

Prospective observational design. Patients had their asthma control, based on symptoms, use of medication, spirometry, and exhaled nitric oxide [F_E_NO], assessed every four weeks during 2^nd^ and 3^rd^ trimester of pregnancy; data on tobacco exposure were also collected prospectively. The primary outcome was episodes of uncontrolled and partly controlled asthma during pregnancy (defined according to GINA-guidelines).

**Results:**

A total of 500 pregnant women with asthma (mean age 30.8 years, range 17 to 44) were consecutively included, of whom 32 (6.4%), 115 (23.0%) and 353 (70.6%), respectively, were current smokers, ex-smokers and never smokers [NS]. Sixty-five NS (18.4%) reported passive tobacco exposure. NS with passive tobacco exposure had significantly lower FEV_1_% predicted (p<0.02) and F_E_NO (p = 0.01) compared to NS without passive tobacco exposure. The relative risk [RR] of an episode of uncontrolled asthma during pregnancy was 4.5 (95% CI 2.7–7.5: p<0.001) in current and ex-smokers compared with never smokers, and 2.9 (95% CI 1.4–5.9; p = 0.004) in NS-women with passive tobacco exposure compared with NS-women not reporting passive tobacco exposure. Treatment with inhaled corticosteroids, most likely as a marker of more severe asthma, was also associated with a higher risk (RR 8.1, 95% CI 5.1–13.0; p<0.001) of an episode of uncontrolled asthma.

**Conclusion:**

Passive tobacco exposure in never smokers is associated with an increased risk of episodes of uncontrolled asthma during pregnancy, which is likely to have adverse effects on pregnancy outcome.

## Introduction

Asthma is one of the most common chronic diseases among fertile women [1]. Previous studies have shown that women with asthma have a higher risk of complications related to pregnancy and birth than women without asthma [2]. Furthermore, studies assessing the impact of asthma on pregnancy outcomes have clearly outlined the importance of best possible asthma control during pregnancy [2], [3]. Maternal asthma has been associated with congenital malformations [4], and poorly controlled asthma appears to be associated with an increase in the incidence and severity of malformations [3].

Tobacco smoking has negative impact on medication requirements and response to pharmacological asthma therapy, which is likely to result in suboptimal asthma control [5], [6]. Furthermore, tobacco smoking, in general, is the single factor that most negatively affects pregnancy in the western part of the world [7].

To date, little is known about the significance of active and passive tobacco smoking on asthma control during pregnancy [7]–[9]. Newman et al. [8] have previously reported that active smoking during pregnancy is associated with an increase in asthma symptoms, whereas no association was reported between asthma symptoms and passive tobacco exposure. However, further evidence is needed regarding the effect of active and passive tobacco exposure during pregnancy on asthma control, also for the purpose of being able to provide the best possible advice to pregnant women with asthma exposed to tobacco smoke.

The aim of this study was, therefore, to investigate the effects of current and former active smoking as well as passive smoking on asthma control during pregnancy.

## Material and Methods

### Material

Since 2007, when the Management of Asthma during Pregnancy (MAP study) was initiated, all pregnant women referred to Hvidovre Hospital (7.000/year, corresponding to 10% of babies born in Denmark every year) have, in a welcome letter from the Department of Gynecology and Obstetrics, been offered participation in the study. Pregnant women accepting the invitation were included in the study provided they filled the following criteria: ^1)^ Asthma, defined according to GINA guidelines (1), ^2)^ At least step 1 asthma therapy, i.e. rescue bronchodilator (1), ^3)^ Visit 1 at the outpatient clinic at the Department of Respiratory Medicine within the first 18 weeks of pregnancy, and ^4)^ Follow-up visit at the outpatient clinic 3 months (+/− one month) after delivery.

## Methods

### 

#### *Characteristics of participants*

Information of age, duration of asthma, current treatment, known allergies and allergic symptoms, smoking status, daily tobacco consumption, duration of smoking and passive tobacco exposure, body weight, and asthma control was collected at the first visit to the outpatient clinic; and follow-up information was collected at each of the following visits. The degree of asthma control was assessed at each visit on the basis of weekly daytime symptoms, weekly nighttime symptoms, weekly use of rescue bronchodilator (i.e. short acting β_2-_agonists), spirometry, and exhaled nitric oxide [F_E_NO]. All women were scheduled for regular follow-up visits approximately every 4 weeks during pregnancy and again 3 months postpartum.

#### *Fractional exhaled nitric oxide measurement*

Fractional exhaled nitric oxide (F_E_NO) was measured at a controlled flow rate of 50 ml/s using an Aerocrine NIOX Flex (Solan, Sweden). The mean value of two measurements in parts per billion (ppb) was registered [10].

#### *Spirometry*

Spirometry, i.e. measurements of FEV_1_ and FVC, was done using an EasyOne Ultrasonic spirometer (Vitalograph, Buckingham, UK). Each test consisted of at least three acceptable FVC maneuvers, and acceptable repeatability was defined as a difference between largest FVC or FEV_1_≤150 ml. The largest FEV_1_ and FVC from at least three acceptable forced expiratory curves were recorded [11].

## Definitions and statistical analyses

Smoking status was defined as *never smoker*, *ex-smoker* (stopped before or when the current pregnancy became known) and *smoker* (minimum 1 cigarette each day); and number of pack-years were calculated as a measure of lifetime tobacco exposure. As the majority of ex-smokers stopped smoking when the current pregnancy became known, current smokers and ex-smokers were grouped together as ever smokers. Passive smoking was defined as living with someone who smokes at home (as smoking in work places, restaurants etc. is not allowed in Denmark).

Partly controlled asthma is defined as daytime symptoms more than twice a week or any limitation of activities or any nocturnal symptoms or awakening or need for rescue inhaler more than twice a week and/or FEV_1_<80% predicted [1]. Uncontrolled asthma is defined as three or more features of partly controlled asthma [1].

The characteristics of groups of interest, e.g. ever smokers, never smokers, and never smokers with passive tobacco exposure, were compared, using either the χ^2^-test or two-sample t-test, as appropriate. The primary outcome variable was episodes of uncontrolled or partly controlled asthma during pregnancy. Factors of possible importance for the primary outcome were entered into a multiple logistic regression model and non-significant variables were deleted by backward elimination to determine those associated with episodes of uncontrolled or partly controlled asthma. A p-value≤0.05 was considered significant. Data was analysed using the SPSS software version 19.0 (SPSS Inc. Chicago, IL, USA).

## Ethics statement

This study was performed in accordance with the Helsinki II declaration, and the study, including procedures related to obtaining written informed consent (obtained from all participants), was according to Danish legislation, incl. for participants below 18 years, and approved by the Research Ethics Committee of the Capital Region of Denmark (H-D-2007-0051).

## Results

### Baseline characteristics of participants

Of the 500 women consecutively included in the study, 353 women were never smokers (70.6%), 115 women were ex-smokers (23.0%), 32 women were current smokers (6.4%). Sixty-five of the never smokers reported passive tobacco exposure (18.4%).

At the time of enrollment, mean age was 30.8 years (range 17 to 44 years) and duration of asthma 12 years (range 0 to 40 years); mean estimated lifetime tobacco exposure for ever smokers was 8.9 pack-years (range 0.5 to 40 pack-years). A total of 167 patients (33.4%) were only treated with prn short-acting β_2_-agonist (SABA), whereas the remaining 333 patients were prescribed inhaled corticosteroids (ICS). Of the 333 patients prescribed ICS, 96 patients were prescribed fixed combination therapy with ICS + long-acting β_2_-agonist (LABA). Only four patients in the cohort were treated with a leukotriene antagonist (n = 4).

Characteristics of never smokers with and without passive tobacco exposure and ever smokers are shown in Table 1. Ever smokers had significantly lower FEV_1%_ predicted compared to never smokers (p<0.005). Furthermore, ever smokers also had significantly lower F_E_NO values compared to never smokers (p = 0.01) (Table 1). Comparing the two groups of never smokers revealed that women with passive tobacco exposure had lower FEV_1_ (p<0.02), F_E_NO (p = 0.01) and were treated with a higher mean dose of ICS (p = 0.01) compared to women without passive tobacco exposure.

**Table 1:** Characteristics, including spirometry, exhaled nitric oxide and prescribed therapy at the baseline visit for 500 consecutively enrolled pregnant women with asthma according to tobacco exposure.

	Ever smokers (ES^1^+ CS^2^) (n = 147)	Never smokers without ETS^3^ (n = 288)	Never smokers with ETS^3^ (n = 65)	NS without ETS vs. NS with ETS
Age, years	29.0 (6.7)	30.8 (4.5)	27.9 (6.4)	<0.05
FEV_1%_ pred.	83.3 (13.3)	91.1 (13.1)	86.7 (13.2)	<0.02
FEV_1_/FVC	0.80 (12.6)	0.82 (0.07)	0.81 (11.9)	Ns
F_E_NO (ppb)	12.4 (12.6)	22.1 (221.0)	15.5 (14.9)	= 0.01
ICS^4^ (µg/day) (n = 333)	444 (180)	302 (195)	392 (205)	= 0.01

Mean values (standard deviation).

1 ES Ex-smokers,

2 CS current smokers,

3 ETS environmental tobacco exposure, and ^4^ICS inhaled corticosteroids.

### Incidence of partly or uncontrolled asthma during pregnancy

A total of 207 (41.6%) of the 500 pregnant women had at least one episode of partly or uncontrolled asthma during pregnancy, and the incidence was 62.6% and 32.7%, respectively, among ever smokers compared to never smokers (p<0.0001). Among never smokers, 55.4% of the women reporting passive tobacco exposure experienced at least one episode of partly or uncontrolled asthma during pregnancy compared to 27.8% among women not reporting passive tobacco exposure (p<0.0001).

### Factors associated with asthma control during pregnancy

The odds ratio (OR) for at least one episode of poorly controlled or uncontrolled asthma during pregnancy was 4.5 (95% confidence interval (CI) 2.7–7.5: p<0.001) for ever smokers compared to the group of never smokers not reporting passive tobacco exposure (reference category) (Table 2). Never smokers reporting passive tobacco exposure had an odds ratio of 2.9 (95% CI1.4 to 5.9; p = 0.004) for at least one episode of partly controlled or uncontrolled asthma during pregnancy compared to the reference category (Table 2). Being prescribed maintenance therapy with inhaled corticosteroids was found to be associated with a higher risk of an episode of uncontrolled asthma during pregnancy (OR 8.1, 95% CI 5.1–13.0; p<0.001). No significant association was found between age, duration of asthma, BMI (based on height and self-reported pre-pregnancy body weight) and allergy, and the risk of an episode of uncontrolled or partly controlled asthma. Furthermore, re-analyzing the data including treatment step as prn SABA, SABA + ICS, and SABA + fixed combination therapy with ICS + LABA (FTC), as a marker of asthma severity, did not reveal any difference in risk for an episode of uncontrolled asthma during pregnancy between those prescribed ICS monotherapy compared to those prescribed FCT. No significant interaction was found between asthma severity, as indicated by treatment step, and tobacco exposure (active or passive).

**Table 2:** Factors predicting an episode of uncontrolled or partly controlled asthma during pregnancy in 500 women.

	Odds ratio (95% confidence interval)	
Never smokers without passive tobacco exposure (n = 288)	Reference	
Current and ex-smokers (n = 147)	4.5 (2.7 to 7.5)	p<0.001
Never smokers with passive tobacco exposure (n = 65)	2.9 (1.4 to 5.9)	p = 0.004
Prescribed inhaled corticosteroids (n = 333)	8.1 (5.1 to 13.0)	p<0.0001

## Discussion

The present prospective study of 500 pregnancies in women with asthma showed, in accordance with previous studies, that former and current active smoking is associated with a higher risk of uncontrolled asthma during pregnancy. However, even more importantly, our study also, and to our knowledge as the first study, showed that passive smoking significantly increases the risk of an episode of uncontrolled asthma during pregnancy.

Examinations and medical advice during pregnancy often naturally focus on the health and lifestyle of the mother. The effect of passive tobacco smoking on asthma control found in this study underlines the importance of advising not only women but also men both before and during pregnancy as the origin of passive tobacco in many cases will be the father-to-be, at least in countries like Denmark where smoking is not allowed in work places, restaurants and public places in general. Our findings therefore strongly support the conclusions in a recently published meta-analysis by Been et al. [12].

Our results concerning active smoking and the risk of uncontrolled asthma during pregnancy are in accordance with findings by Murphy et al. [6] stating that asthma exacerbations are more common and more severe in current smokers than never smokers during pregnancy. In line with this, our findings support a recent study by Saba et al. [13] reporting the need for evidence-based smoking cessation programs designed and tailored to assist asthma patients who smoke to quit smoking. This is also shown by Newman et al. [8] who reported that active smoking among pregnant women with asthma is associated with increased asthma symptoms. However, the study by Newman et al. [8] reported, in contrast to our study, no differences in symptom exacerbation between nonsmokers with and without passive household smoke exposure also defined as living with someone who smokes at home reported in a questionnaire. This difference in observations may be due to selection of participants and, not least, the method used for collection of data on passive tobacco exposure, as the participants in the present study were interviewed about passive tobacco exposure at each of the every 4-weeks visits to the outpatient clinic.

We found a significant association between being prescribed maintenance therapy with inhaled corticosteroids and risk of uncontrolled asthma during pregnancy. The patients prescribed controller therapy is likely to have more severe asthma [1] compared to patients only treated with as needed bronchodilator, which is most likely the explanation for the observed association. Furthermore, we also observed what might be interpreted as a dose-response relationship between smoking status and daily prescribed dose of inhaled corticosteroid, which is in keeping with previous findings by Chalmers et al. [14], as they documented the reduced efficacy of inhaled corticosteroid therapy in asthma patients exposed to tobacco smoke.

In the present study, no significant relation was found between allergy, BMI, and the risk of an episode of uncontrolled asthma. IgE-mediated allergy causing symptoms of allergic rhinitis may be assumed to worsen symptoms of asthma. However, patients suffering from intrinsic, i.e. non-allergic asthma, are often more difficult to treat and control [15], [16] and this may explain the observed lack of association. Obesity is known to have negative impact on asthma control and response to therapy [17], [18]. However, we found no association between BMI and risk of uncontrolled asthma, probably due to the substantial impact of tobacco exposure on asthma control [5]; although, the lack of association might also be related to the relatively low mean BMI among the enrolled women.

In conclusion, the present study revealed not only an association between former and current active smoking and episodes of uncontrolled asthma during pregnancy, but also that passive tobacco exposure is a risk factor for episodes of uncontrolled asthma during pregnancy. Not least the latter association, to our knowledge not previously reported, should be made known to all women with asthma in the reproductive age due to the known association between uncontrolled asthma during pregnancy and adverse pregnancy outcomes.
